# HIV infection early diagnosis experience in primary care

**DOI:** 10.7448/IAS.17.4.19597

**Published:** 2014-11-02

**Authors:** Francisco Jover Diaz, Paz Ortega, Pedro Antequera, Blas Cloquell, Marta Alcaraz, Mavi Hernandis, Carlos Nuñez, Rosario Lloret, Faustino Perez, Sabina Jover Perez, Fernando Buñuel, Francisco Gomez, Marta Sanz, Rafael Ordovas, Francisco Torregrosa, Ángela Barceló, Consuelo Masegosa, Victoria Ortiz de la Tabla, Jose María Cuadrado

**Affiliations:** 1 Infectious Diseases, Hospital Clinico San Juan Alicante, Alicante, Spain; 2 Hospital Clinico San Juan Alicante, CSI C/Gerona, Alicante, Spain; 3 Microbiology, Hospital Clinico San Juan Alicante, Alicante, Spain; 4 Hospital Clinico San Juan Alicante, CS El Cabo, Alicante, Spain; 5 Hospital Clinico San Juan Alicante, CS Sta Faz, Alicante, Spain; 6 Hospital Clinico San Juan Alicante, CS Juan XXIII, Alicante, Spain

## Abstract

**Introduction:**

Traditional screening system focus on classic risk factors “lost” a substantial proportion of HIV-infected patients. Several organizations such as CDC or USPS Task Force favour universal screening for HIV infection for good cost-effectiveness profile. In a previous study prevalence of HIV infection in patients attending our infectious diseases department was high (5.4%).

**Objective:**

To determine prevalence of HIV infection in patients aged 20–55 years in primary care (PC).

**Material and Methods:**

A propsective observational study was undertaken between February and June 2013. We performed a screening of HIV infection type “Opt-out” (offering voluntary rejection) in 4 PC centers (32 Physicians) in San Juan-Alicante. Sample size (n=318) for a prevalence of 1% and a confidence level of 97% was calculated. Nevertheless, other PC physician not recruiting patients performed HIV testing according clinical risk factors.

**Results:**

HIV testing was offered to 508 patients. Mean age 38.9±10 years (58.5% female). Overall, 430 (83.8%) agreed to participate. Finally, 368 patients (71.7% of total) were tested for HIV. No patient had a positive result (100% ELISA HIV negative). However, following clinical practice, 3 patients were diagnosed of HIV in the same period by non-recruiting physicians. In 2 cases, serology was performed at the patient's request and in one case by constitutional syndrome. The 3 patients were MSM.

**Conclusions:**

1) In our study, we detected no new cases of HIV infection through universal screening. 2) Our screened population could be lower-risk because of high percentage of women included (58.5%). 3) Performing HIV opt-in screening (clinical practice), we detected 3 cases in the same period, all having HIV risk factors (MSM). 4) These results suggest that opt-out screening should be developed in high-risk populations. It is still to be determined what is the best screening strategy in low-risk populations such as ours.

